# Influence of two consecutive partial lateral corpectomies on passive motion of the canine lumbar spine

**DOI:** 10.1111/vsu.70048

**Published:** 2025-12-05

**Authors:** Lisa F. Becker, Robin Heilmann, Stefan Schleifenbaum, Stefan Kohl, Thomas Flegel

**Affiliations:** ^1^ Department for Small Animals, Faculty of Veterinary Medicine Leipzig University Leipzig Germany; ^2^ ZESBO – Center for Research on Musculoskeletal Systems, Department of Orthopaedic Surgery, Traumatology and Plastic Surgery, Faculty of Medicine Leipzig University Leipzig Germany; ^3^ Present address: Tierärzte IVC Evidensia GmbH Tierklinik Lüneburg Lüneburg Germany; ^4^ Present address: Tierärzte IVC Evidensia GmbH Tierklinik Hofheim Hofheim Germany

## Abstract

**Objective:**

To assess the effects of a first and second consecutive partial lateral corpectomy (PLC) on the passive range of motion (ROM) of canine lumbar spinal segments.

**Study design:**

Controlled, ex vivo biomechanical study.

**Sample population:**

Adult canine cadaveric spines (*n* = 10).

**Methods:**

Ten canine lumbar spinal segments were embedded in cast resin and aluminium tubes at their respective ends and attached to a spine testing bench. The ROM was measured under a torque of 2 Nm in three movement directions before and after the first PLC at L2–L3 and the second PLC at L3–L4.

**Results:**

In the sagittal plane, mean ROM increased by 2.4° after the first PLC and by an additional 1.1° after the second PLC. In the dorsal plane, mean ROM increased by 2.3° after a first PLC and by an additional 1.5° after a second PLC (*p* < .05). Differences in mean rotational ROM before and after one or two PLCs were not identified.

**Conclusion:**

Each of the two PLCs resulted in a significant increase in ROM in the sagittal and dorsal planes (*p* < .05). The second PLC did not increase the ROM to a greater extent than the first.

**Clinical impact:**

Performing each of two adjacent PLCs can lead to a reduction in the stability of the lumbar spine in dogs. However, in this study, the destabilizing effect of the second PLC was not greater than the effect of the first PLC.

**Abbreviations**
C: celsius
Cm: centimeter
CT: computed tomography, computed tomography scanner
ECVN: European College of Veterinary Neurology
ESVN: European Society of Veterinary Neurology
et al.: et alia
h: hour, hours
Hz: hertz
i.e.: id est
kg: kilogram
kVp: peak kilovoltage
L: lumbar vertebra
mAs: milliampere seconds
min: minutes
mm: millimeter
n: sample size
Nm: newton meter
NV: naamloze vennootschap, stock corporation
PLC: partial lateral corpectomy
ROM: range of motion
ZESBO: Zentrum zur Erforschung der Stütz‐ und Bewegungsorgane, Center for Research on Musculoskeletal Systems

## 1 INTRODUCTION

Partial lateral corpectomy (PLC), first described in 2004 by Moissonnier et al.,^1^ is the most promising surgical technique to treat chronic, thoracolumbar disc disease, which causes predominant ventral myelocompression. It allows adequate decompression of the spinal cord and minimizes the risk of iatrogenic spinal cord injury.^1^, ^2^ Thoracolumbar PLC creates a lateral slot into two adjacent vertebral bodies and the intervertebral disc between them.^1^ The recommended maximum slot dimensions are one‐quarter of the vertebral body length into each adjacent vertebra (craniocaudal extension), two‐thirds of the vertebral body width (depth), and one‐half of the vertebral body height.¹

In 2012, Vizcaíno Revés et al. showed that performing a single lumbar PLC in canine cadavers led to a significantly increased passive flexion and extension, and lateral bending to both sides.^3^ They therefore recommended further studies before performing multiple PLCs.^3^ Similar findings were reported by de Vicente et al. in 2013,^4^ who observed increased passive lateral bending in a 2013 biomechanical study of single lumbar PLCs in canine cadavers.

Within one patient, single disc herniations occur more frequently than multiple disc herniations.^5^ Nevertheless, neurological deficits can result from multiple herniated discs producing a similar degree of myelocompression.^5^, ^6^, ^7^, ^8^ In these cases, depending on the surgical method used, some authors recommend treating all herniated discs to achieve adequate decompression of the spinal cord.^5^, ^9^ Multiple herniated discs do not always occur at the same time. It is possible that, after surgical treatment of a herniated disc, further herniated discs may occur later and also require surgical treatment.^7^, ^10^ When surgically decompressing several herniated discs, the cumulative destabilizing effects on the spine should be considered.^9^

Given the occasional clinical need to perform multiple PLCs,^5^, ^6^, ^7^, ^8^, ^9^ the finding that even one PLC can lead to a substantial increase in range of motion (ROM) of the spine,^3^, ^4^ and the need for biomechanical studies on multiple PLCs,^3^ this study aimed to investigate the biomechanical consequences of two PLCs performed on two adjacent intervertebral disc spaces on the right side of 10 canine lumbar spinal segments. These biomechanical consequences were investigated ex vivo by measuring changes in passive spinal ROM in three different directions of movement under a torque of 2 Nm, comparing native canine lumbar spinal segments with the same spinal segments after completion of the first and second consecutive PLCs on the right side. We hypothesized that the second PLC would cause a greater increase in passive spinal ROM and thus has a greater impact on spinal stability than the first PLC.

## 2 MATERIAL AND METHODS

### 2.1 Animals

Canine spinal segments L1–L5 were harvested from dogs that died or were euthanized for reasons that were not related to the study and that were not due to spinal diseases, and were donated by their owners. Donor dogs had to fulfill the following criteria in order to be included in this study: patient age between 1 and 12 years; body mass between 20 and 35 kg; absence of clinical signs of a spinal disease before death; absence of pathological changes at L2–L4 based on a computed tomography examination.

Approval to perform the study was obtained from the ethics committee of the Faculty of Veterinary Medicine of Leipzig University (approval number: EK 11/2021).

### 2.2 Spinal imaging

A lumbar spinal computed tomography (CT) scan of all spines was performed at two different times, using a 128‐slice spectral CT (Philips IQon Spectral CT, Koninklijke Philips NV, Amsterdam, Netherlands). The first scan was performed to confirm the absence of lumbar pathology before biomechanical testing. The second scan was conducted after biomechanical testing to ensure that the dimensions of the slots matched the planned dimensions and to identify possible tissue damage. The following scan parameters were used: helical acquisition, 0.8 to 0.9 mm slice thickness, 0.4 to 0.45 mm overlap, 512 × 512 matrix size, 78–88 mm field of view, tube voltage of 120 to 140 kVp, and tube current of 100 to 250 mAs, depending on the patient's size. Images were reconstructed using a bone and soft tissue kernel and evaluated for possible spinal pathologies before, and tissue damage after, biomechanical testing on a dedicated workstation with multiplanar reconstruction by two investigators in consensus.

### 2.3 Spinal segment preparation and embedding

Each spinal segment (L1–L5) was removed within 48 h after the donor dog died or was euthanized. The spinal segments were harvested, sparing the intervertebral discs L1 to L5, interspinal ligaments, and the intervertebral joints. All other paraspinal structures were removed. Following preparation, the spinal segments were wrapped in physiological saline‐soaked cotton towels and frozen at −18°C until the day of biomechanical testing.

All frozen spinal segments were thawed to room temperature, starting 48 h before biomechanical testing. After thawing, a 5 cm long and 3.5 mm diameter wood screw was placed transversally through the cranial half of the vertebral body of L1 and another one through the caudal half of the vertebral body of L5. In its final position, the screw protruded approximately 1 cm from each vertebral body. The screws were intended to increase adherence between the spinal segment and the embedding material. Each spinal segment was embedded at its respective ends (L1 and the cranial half of L2; L5 and the caudal half of L4) in 15 cm diameter, 5 cm high aluminium tubes using cast resin, establishing a connection between the spinal segments and the testing bench. The cast resin consisted of isocyanate (Rencast FC 52/53 isocyanate, Huntsman Corporation, Salt Lake City, Utah), polyol (Rencast FC 52 polyol, Huntsman Corporation), and a filler material containing aluminium hydroxide (DT 082–1 Füller, Gößl & Pfaff GmbH, Karlskron, Germany). While fixing the spinal segment's cranial end using a tripod, its caudal end was orientated in the aluminium tube so that the dorsal spinal process pointed in the direction of the tripod and the floor of the spinal canal was centered in the middle of the aluminium tube. Cast resin was then poured into the aluminium tube until it covered the entire L5 vertebra and the caudal half of L4. The same embedding procedure was used for fixation of the cranial end of the spinal segments, embedding the entire L1 vertebra and the cranial part of L2 vertebra, after a curing period of 30 min.

### 2.4 Biomechanical testing

**FIGURE 1:**
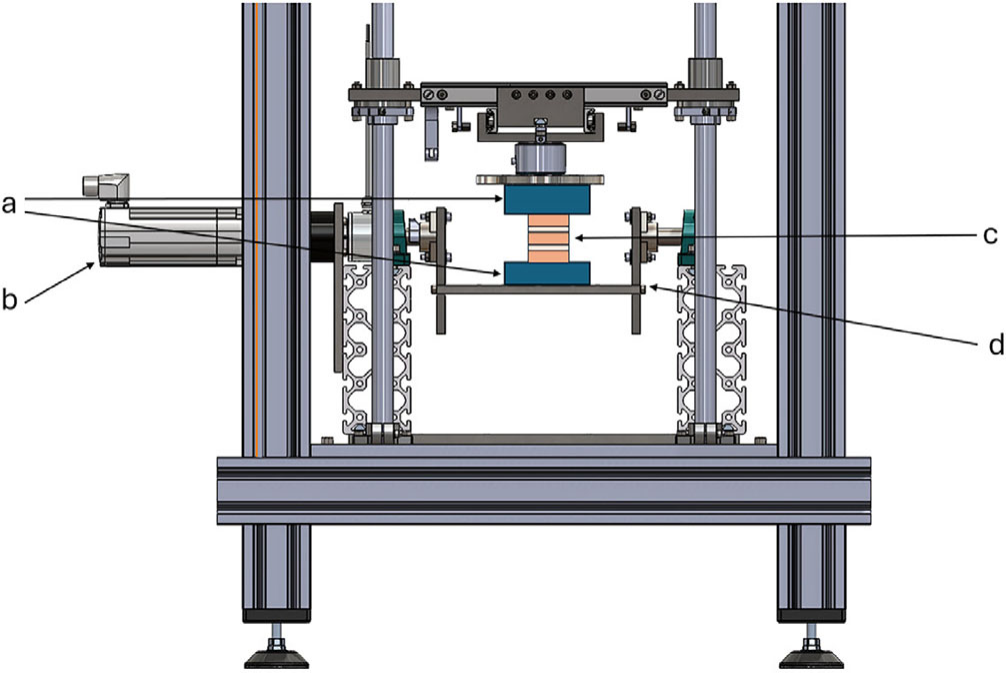
Spine testing bench for testing extension, flexion, and lateral bending. The spinal segments (c), embedded in cast resin and aluminium tubes (a), were moved with the aid of a servomotor (b) using a bidirectionally movable bottom plate (d). Extension, flexion, and lateral bending were evaluated using this setup.

**FIGURE 2:**
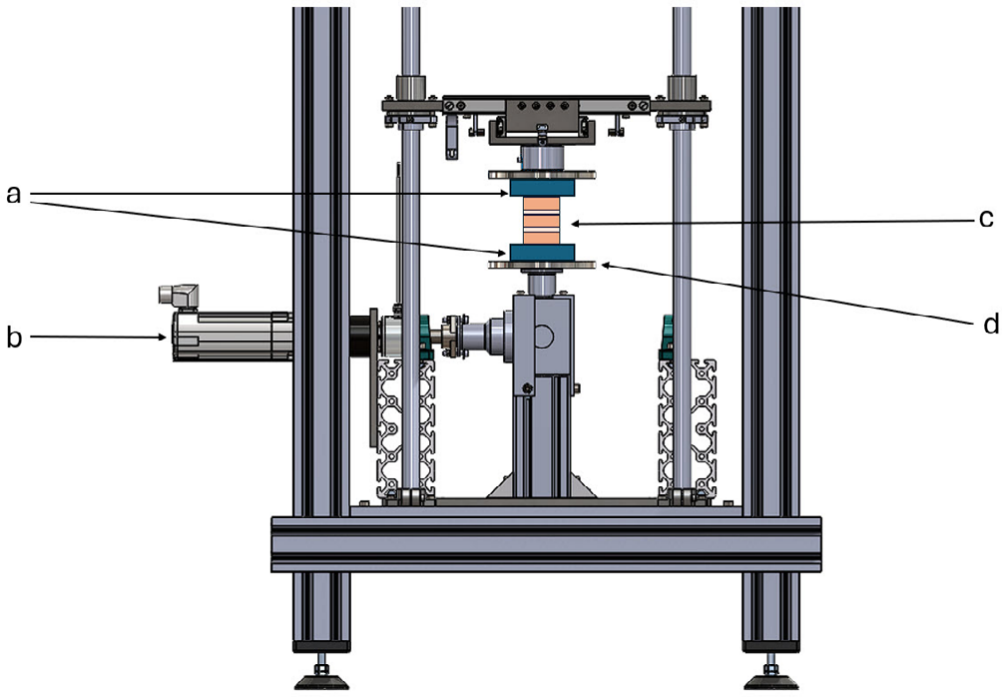
Spine testing bench for testing rotation. The spinal segments (c) embedded in cast resin and aluminum tubes (a) were moved with the aid of a servomotor (b) using a clockwise and counterclockwise movable plate (d). Only rotation was tested in this setting.

The spine‐testing bench, described by Schleifenbaum et al.,^11^ was modified for this study's purposes. Each spinal segment was fixed on the testing bench in a vertical orientation. The aluminium tube, including the cranial spinal segment's end (L1–L2) pointing upwards, was stationary fixed to the bench, whereas the aluminium tube including the caudal spinal segment's end (L4–L5) was attached to a bidirectionally moveable bottom plate (Figure 1 and Figure 2). Segments fixed in that way could be moved in two opposing directions by a servo motor (BMH0703T01A2A, Schneider Electric GmbH, Rueil‐Malmaison, France) and a gearbox (GBX060008K, Schneider Electric GmbH) with a frequency of 0.5 Hz and a torque of 2 Nm. An integrated protractor allowed the bending angle (representing the passive ROM) to be measured.

Before the study examinations, a control measurement was performed on one native spinal segment to determine the influence of repeated biomechanical testing itself on the passive spinal ROM. The segment was examined using the same protocol as the planned biomechanical tests but without performing any PLCs.

Ten spinal segments were tested in their intact state before surgical intervention (first test series), after a single PLC between L2 and L3 from the right side (second test series), and after a second PLC between L3 and L4 from the right side (third test series). In each series, all spinal segments underwent the following tests with a torque of 2 Nm applied to their caudal parts: 10 movement cycles within the sagittal plane, 10 within the dorsal plane, and 10 within the transverse plane. One movement cycle was defined as the total passive ROM, i.e., the motion between the two maximum angles, either between maximum dorsal extension and ventral flexion, or maximum bending to the left and right, or maximum rotation to both sides. Within the sagittal plane, extension and flexion were also measured individually and compared with each other. Comparative measurements were also made contrasting left and right lateral bending within the dorsal plane and contrasting rotation to the left and right within the transverse plane. Movement cycles 1 to 5 were used for tissue preconditioning, and cycles 6 to 10 were used for statistical analysis.

To perform PLC, each spinal segment was released temporarily from the spine testing bench. Drilling was performed freehand using the Midas Rex Legend Electric System (Medtronic GmbH, Meerbusch, Germany) and a 5 mm rose head bur. For each segment, the anticipated PLC slot dimensions were determined according to the recommendations of Moissonnier et al.^1^ A depth gauge was used to verify whether the planned slot dimensions were achieved.

An additional spinal segment, meeting all inclusion criteria, underwent identical testing but did not undergo any PLC. After having performed the three different testing series for measuring the ROM, this unmodified spinal segment, as well as one randomly selected spinal segment, which had undergone two PLCs, were used for failure testing in left and right lateral bending. Torque was increased in 1 Nm steps, starting with 3 Nm, until the segment was destroyed or the spine testing bench's maximum torque capacity of 25 Nm was reached. Ten movement cycles were performed at any torque step.

Data were aggregated using Excel for Mac (Version 16.47, Microsoft Corporation, Redmond, Washington). Increases in ROM were expressed as absolute angles and percentage. Statistical analysis was performed with SPSS Statistics 27 (IBM Corporation, Armonk, New York). Differences in ROM between groups were tested using the Friedman test and the Wilcoxon signed‐rank test, with the significance level set at *p* < .05. If the Friedman test did not detect any significant differences among the three different test series, the corresponding measurements were excluded from further analysis using the Wilcoxon signed‐rank test.

## 3 RESULTS

Eleven out of 18 selected lumbar spinal segments were included in this study, and seven segments were discarded because of osseous pathological changes in the lumbar spine identified on the first CT. Lumbar spines of the following breeds were included: Labrador retriever (*n* = 4) and one of each of the following breeds: Bernese mountain dog, bull terrier, standard schnauzer, German shepherd, Australian shepherd, basset griffon, and German longhaired pointer. Seven dogs were female, three of which were spayed. Three dogs were male, none of which was neutered.

### 3.1 Nondestructive tests

Ranges of motion within the sagittal plane were analyzed by comparing extension and flexion, and within the dorsal plane by comparing left and right lateral bending. In flexion, ROM was 7.7° ± 1.0° (first test series, before PLC), 9.6° ± 1.1° (second test series, after one PLC), and 10° ± 1.3° (third test series, after two PLCs). In extension, ROM was 5° ± 2.5° (first test series), 5.5° ± 2.3° (second test series), and 6.2° ± 2.9° (third test series). Across all three test series, flexion consistently produced greater ROMs than extension, with differences of *p* < .05 in every case. Specifically, *p* = .022 for comparisons between extension and flexion in the first and second test series, and *p* = .017 for the third test series.

In the dorsal plane, right lateral bending (toward the PLC) measured 13.7° ± 2.3° (first test series), 15.4° ± 2° (second test series), and 16.3° ± 2.2° (third test series), whereas left lateral bending measured 12.1° ± 1.4° (first test series), 12.7° ± 1.6° (second test series), and 13.3° ± 2° (third test series). Differences between left and right lateral bending were observed in the second (*p* = .005) and third (*p* = .017) test series, i.e., after performing the PLCs. In these series, right lateral bending toward the PLC was consistently greater than left lateral bending.

**FIGURE 3:**
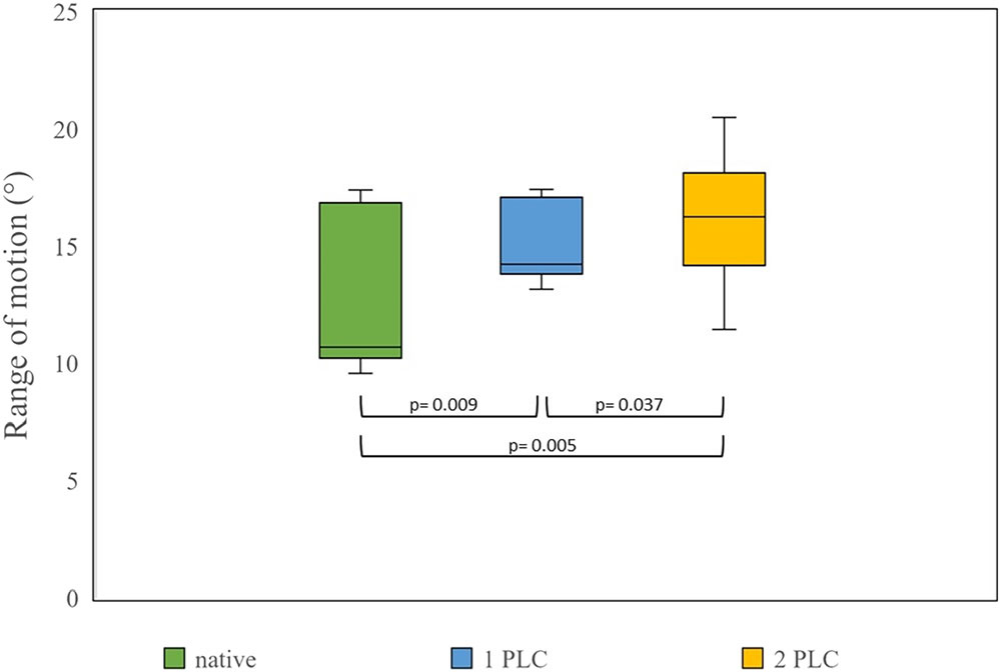
Range of motion (ROM) during extension and flexion was compared between lumbar spinal segments in their native condition and after performing the first and second partial lateral corpectomies (PLC) (*n* = 10 lumbar spinal segments). Boxes represent the 50% interquartile range, with the line inside each box indicating the median ROM for each test series. Whiskers represent all measurements that are smaller than the first (lower whisker) or greater than the third (upper whisker) quartile. The curved line beneath the boxes indicates significant differences between the test series.

In the sagittal plane, the mean range of motion (ROM) between the dorsal and ventral extremes was 12.7° ± 3.1° in the intact spine, 15.1° ± 1.6° after the first PLC, and 16.1° ± 2.6° after the second PLC (Figure 3). When expressed as percentages, with 100% defined as the ROM of the intact spine, the mean sagittal ROM increased to 123% and 130% after the first and second PLC, respectively.

**FIGURE 4:**
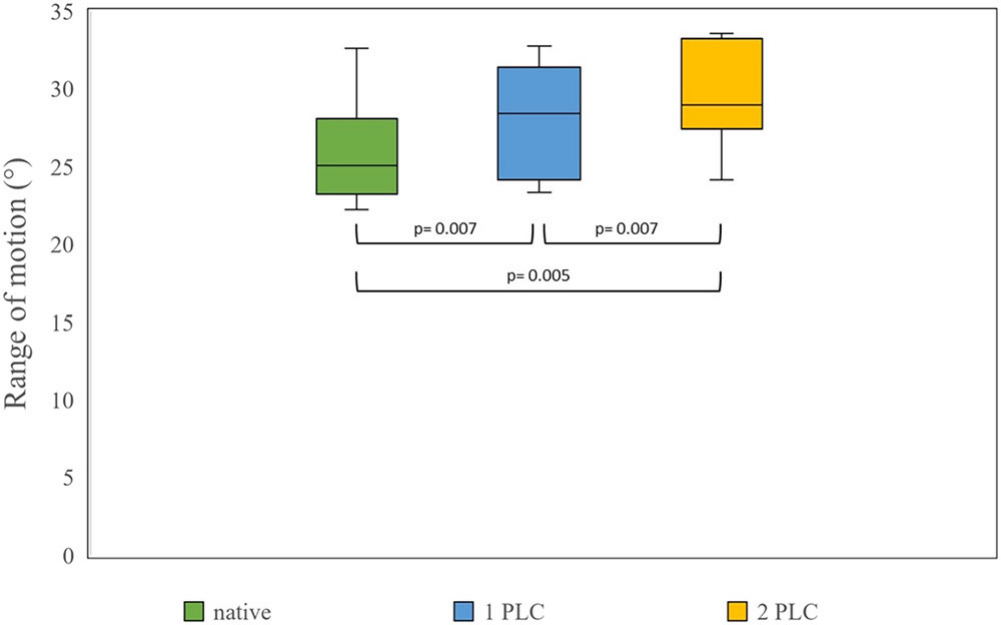
Range of motion (ROM) during left and right lateral bending was compared between lumbar spinal segments in their native condition and after performing the first and second partial lateral corpectomies (PLC) (*n* = 10 lumbar spinal segments). Boxes represent the 50% interquartile range, with the line inside each box indicating the median ROM for each test series. Whiskers represent all measurements smaller than the first (lower whisker) or greater than the third (upper whisker) quartile. The curved line beneath the boxes indicates significant differences between the test series.

In the dorsal plane the mean ROM between the left and right extremes was 25.8° ± 3.0° in the intact spine and increased to 28.1° ± 3.2° and to 29.6° ± 3.4° after the first and second PLC, respectively (Figure 4). Expressed as percentages, the mean dorsal ROM was 109% after the first PLC and 115% after the second PLC relative to the intact spine.

**FIGURE 5:**
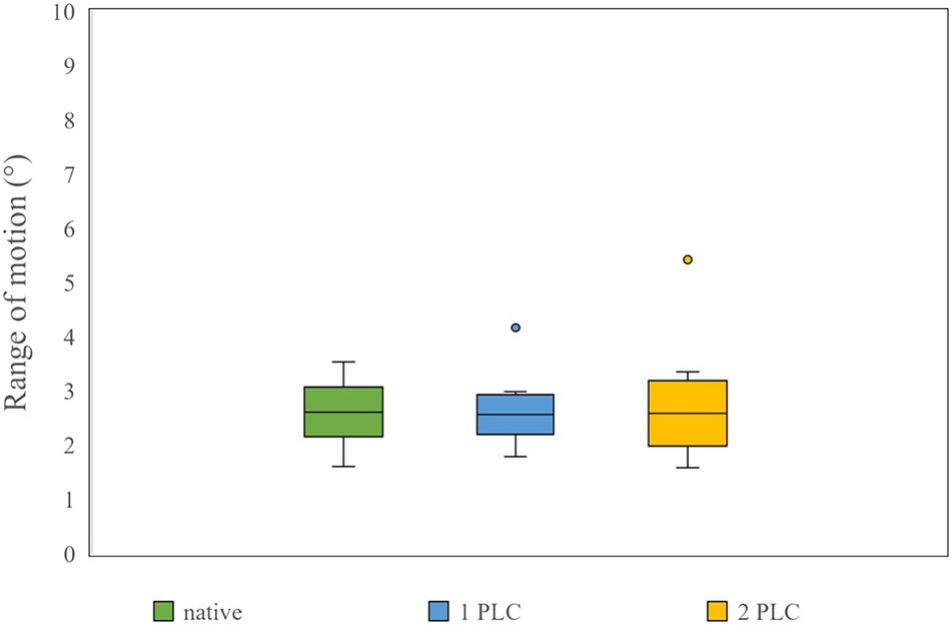
Range of motion (ROM) during left and right rotation was compared between lumbar spinal segments in their native condition and after performing the first and second partial lateral corpectomies (PLC) (*n* = 10 lumbar spinal segments). Boxes represent the 50% interquartile range, with the line inside each box indicating the median ROM for each test series. Whiskers represent measurements smaller than the first (lower whisker) or greater than the third (upper whisker) quartile, but within 1.5 times the interquartile range from the respective quartile. Outlier values, depicted as dots, lie more than 1.5 times the interquartile range away from the box.

The mean ROM in rotation between the left and right extremes was 2.6° ± 0.6° in the intact spine, 2.7° ± 1.0° after the first PLC, and 2.8° ± 1.0° after the second PLC (Figure 5). When expressed as percentages, the mean total ROM was 102% after the first PLC and 106% after the second PLC, relative to the intact spine.

Based on the Friedman test, differences in ROM were observed among the three test series in the sagittal plane, with extension and flexion as the respective extremes (*p* < .001). In the dorsal plane, with maximum left and right lateral bending as extremes, the Friedman test also revealed differences in ROM among the three test series (*p* < .001). In contrast, no differences in ROM were observed for rotation to the left and right between any test series (*p* = .5). Rotation to the left and right was therefore excluded from further statistical analysis.

The Wilcoxon signed rank test was used to compare the ROM in paired measurements within each movement direction in the sagittal and dorsal planes. Based on this test, while evaluating extension and flexion the increase in ROM after the first PLC was substantial (*p* = .009), as was the increase in ROM when comparing the second (after 1 PLC) and third (after 2 PLCs) test series (*p* = .037), and comparing the first and third test series (*p* = .005).

Again, based on the results of the Wilcoxon signed rank test, ROM in lateral bending increased after the first PLC (*p* = .007), and also increased when comparing the second and third test series (*p* = .007) and the first and third test series (*p* = .005). In both the sagittal plane, with extension and flexion as the corresponding extremes, and the dorsal plane, with left and right lateral bending as the corresponding extremes, the increase in ROM after the second PLC disruption was not greater than that after the first PLC disruption (*p* > .05).

Based on the Wilcoxon signed rank test, the corresponding increases in ROM between the first and second test series, and between the second and third test series, did not differ, with *p* = .2 for the sagittal plane and *p* = .3 for the dorsal plane.

### 3.2 Outcome of control testing and post‐mechanical‐testing CT scan

Comparing all measurements from the control tests, only small fluctuations were observed, which were smaller than the variations arising from testing the ROM of 10 different spinal segments within one direction of movement and one test series. The following ROMs were measured during three test cycles: 13.5° ± 0.1°, 13.6° ± 0.1°, and 13.9° ± 0.1° in extension and flexion; 24.1° ± 0.1°, 24.7° ± 0.0°, and 23.6° ± 0.4° in left and right lateral bending; and 1.7° ± 0.0° in rotation to the left and right, measured three times. A second CT scan was performed on all spinal segments after completing the three biomechanical test series to evaluate any structural changes caused by the test procedure itself. No tissue damage was seen in any segment.

## 4 DISCUSSION

In the context of chronic disc herniation, surgical removal of the herniated disc material is often challenging because the disc material is very hard, encapsulated, and tends to have ventral adhesions to the dura mater and other extradural structures.^1^, ^12^ The release of the adhesions, in particular, can result in more intensive manipulation of the spinal cord if the goal of complete decompression of the spinal cord is pursued intraoperatively.^13^ This intensive spinal manipulation can cause a temporary deterioration in the neurological condition of affected patients immediately postoperatively.^13^

For a long time, Hansen Type 2 disc protrusions were not surgically decompressed because of these difficulties in removing the protruded disc material.^1^, ^14^, ^15^, ^16^ To minimize the risk of iatrogenic damage to the spinal cord while achieving adequate decompression without excessive bone removal, Moissonnier et al. developed the PLC.^1^ By localizing the surgical slot exactly at the level of the intervertebral disc, the disc is fenestrated intraoperatively without additional surgical effort.^1^ This approach also minimizes the risk of recurrence within the affected intervertebral disc space.^2^ The most common patients to be treated with a PLC are older large‐breed dogs and dachshunds.^2^, ^12^, ^13^ Despite its advantages, this surgical method can alter the biomechanical properties and stability of the spine.^3^, ^4^, ^12^

The aim of this study was to assess the influence of two consecutive PLCs on passive spinal ROM applying a torque of 2 Nm. We were able to show that each of the two PLCs increased a spinal segment's ROM in the sagittal and dorsal planes. Comparing the influence of the first and second PLC on the passive spinal motion in the sagittal plane after the first PLC, an increase of 2.4° (i.e., 23% of passive spinal ROM in native condition) was identified. After the second PLC an additional increase of 1.0° (i.e., 8% of passive spinal ROM in native condition) was measured. In the dorsal plane an increase of 2.3° (i.e., 9% of passive spinal ROM in native condition) was detected after the first PLC and an additional increase in passive spinal motion of 1.5° (i.e., 6% of passive spinal ROM in native condition) was detected after the second PLC. The respective influence of the first and second PLC on the passive spinal ROM did not differ within the sagittal and dorsal planes (*p* > .05). Contrary to our original hypothesis, the second PLC had no greater influence on passive spinal ROM than the first.

The absence of a greater effect of the second PLC on passive spinal motion compared with the first PLC may be explained by the spine's passive stabilizing system. This system consists of spinal ligaments, intervertebral discs and joint capsules and develops its reactive forces only at the respective spinal limits of resilience against tensile forces.^17^ This allows spinal columns to resist deformation by becoming more rigid under increasing loads while being flexible towards light loads.^17^, ^18^ Performing two consecutive PLCs may activate this passive stabilizing system to counteract the increased spinal ROM. Whether the additional insertion of further PLCs would have an ever decreasing effect on the ROM must be clarified through further studies.

Rotation was the only type of movement in this study that was not substantially affected by performing a PLC. This finding is consistent with that of Vizcaíno Revés et al.^3^ For rotational stability, the intervertebral disc is the most important anatomic structure within a single vertebral motion unit.^19^ When the integrity of the intervertebral disc is reduced, for example by performing a PLC, the articular facets, dorsal lamina, and pedicle become the next most important stabilizing structures in rotation.^19^ The fact that these three structures remained intact in this study may therefore explain why two PLCs did not result in a substantial change in ROM. Within one vertebral motion unit of the thoracolumbar spine, the two facet joints account for approximately 10% of spinal stability.^19^ The importance of the facet joints for spinal stability is further supported by the finding that, after discectomy, they are the most vulnerable structures in biomechanical tests of rotational stability.^19^ Using strain gauge testing of canine lumbar facet joints, it has also been shown that the facet joints are subjected to greater loads during torsion and extension than during flexion or lateral bending.^20^, ^21^

Schulz et al. demonstrated that, in biomechanical testing of the T13–L1 motion unit, the articular facets of the canine thoracolumbar spine contribute more to stiffness in axial rotation and dorsoflexion than during lateral bending and ventroflexion.^20^ As the geometric shape of the osseous facets is complex and variable,^22^ and their orientation relative to the long axis of the spine varies both within the same spine and among individuals,^22^ the interlocking effect of the facet joints^23^ during rotation also varies. In the canine lumbar spine, the articular processes from L1 to L7 have been found to exhibit similar geometry.^24^

Spinal stability has been assessed in relation to intervertebral disc pathology since 1944.^18^, ^25^ Most biomechanical studies on spinal stability ultimately address the question of the load at which clinical instability occurs and the factors responsible for its onset. However, no reliable evidence has yet been established regarding the point at which biomechanical destabilization of the spine becomes clinically relevant.

To advance understanding of this issue and to support future investigations, one native spinal segment and one spinal segment with two PLCs were used as examples to investigate the torque required to produce macroscopically visible tissue damage in lateral bending. No visible tissue damage occurred in the unaltered segment up to a torque of 25 Nm, whereas the segment with two PLCs tore in the right intertransverse ligament between the second and third lumbar vertebrae at a torque of 13 Nm.

It is assumed that the physiological load in canine lumbar spines corresponds to a torque of approximately 2 Nm.^3^, ^26^ The torque of 13 Nm at which tissue damage became evident in the spinal segment with two PLCs is therefore unlikely to occur under physiological conditions. Despite the reduced load‐bearing capacity observed after two PLCs, this may explain why two to four PLCs have been performed successfully in individual patients in clinical practice.^2^, ^8^, ^12^

As these failure tests were performed on only one spinal segment at a time, the results do not permit statistical analysis and are subject to individual, nonestimable variability. However, these results should be used as a starting point for further studies.

Several studies have described spinal instability as a condition in which surgical intervention is required because spinal resilience to physiological load is lost and neurological deficits, spinal deformation, and spinal pain manifest clinically.^18^, ^20^, ^27^, ^28^ This view of spinal instability also applies in this study.

As each of the two PLCs led to an increase in ROM in the sagittal and dorsal plane, it can be assumed that spinal resilience was reduced. Whether this reduction in spinal resilience would lead to neurological deficits in vivo remains uncertain.

According to a study by Ferrand et al. on the surgical treatment of chronic intervertebral disc disease using PLC in 107 dogs, vertebral instability occurred in 1.8% of patients (*n* = 2).^12^ One of the two affected patients received PLC alone and another patient was treated surgically with PLC and an additional mini‐hemilaminectomy.^12^ From these results it appears that even a single PLC has the potential to trigger spinal instability.

In addition to biomechanical studies on canine cadaveric spines, finite element analysis is a useful method for more accurately determining the stress level at which spinal resilience is lost. Kikuchi et al. were able to show that mechanical tests on lumbar spinal segments conducted using finite element models give comparable results to biomechanical cadaveric studies.^29^, ^30^

One recent study employed finite element analysis to evaluate the biomechanical effects of different surgical decompression procedures on an L1–L2 spinal segment.^29^ Among other surgical techniques the effects of a PLC and a hemilaminectomy on the spinal segment were examined.^29^ The results indicated that, within the elastic range of the L1–L2 segment, displacement angles under torques of 0.4 to 2 Nm during extension, flexion, lateral bending, and rotation were greater for hemilaminectomies than for PLCs.^29^ Assuming that the probability of spinal failure increases with displacement angle, and considering that the literature reports the safe performance of three to six consecutive PLCs,^3^, ^17^, ^31^, ^32^, ^33^ this suggests that performing up to six PLCs may also be feasible.

In contrast to studies on cadavers or finite element analysis, the paraspinal muscles do have an additional active spine stabilizing effect in vivo.^34^, ^35^, ^36^, ^37^ It can therefore be assumed that the destabilizing effect caused by two consecutive PLCs as examined in this study is smaller in vivo than suggested by the results presented here. The destabilizing effect of a PLC may also decrease over time according to postoperative healing tendencies as fibrocartilage fills in disc defects in vivo.^29^, ^38^, ^39^ Nevertheless, whenever postoperative deterioration in neurological condition persists or whenever spinal pain exceeds normal postoperative levels, careful diagnostic workup, including assessment for spinal instability, should be considered.

This study has limitations. Age has a great impact on the stiffness and flexibility of spinal segments.^20^, ^40^ As the spinal stiffness increases and spinal flexibility decreases with age this study's results may be influenced by the patient's age as patients included in this study were between 1 and 12 years old at their time of death. Results outside that age range may differ from those presented here. It would also have been preferable to have cadaver specimens from young healthy dogs to minimize variation for comparison purposes. Excluding spines with pathological changes in the interest of consistency may also have influenced the results. As only patients with unremarkable CT were included in this study, the effect of disc protrusion on spinal stability, as it appears in clinical patients, could not be evaluated.

In vivo, disc degeneration and protrusion might negatively influence or decrease spinal stability.^17^, ^41^, ^42^ It might therefore be possible that a larger ROM could be seen in patients with degenerated or protruded intervertebral discs. It is common to perform biomechanical spine tests on preparations in which paraspinal musculature has been removed. This is advantageous in order to compare results from different studies but it ignores the stabilizing effect of paraspinal muscles. As biomechanical properties of spinal segments in vitro without paraspinal musculature do not correspond exactly to biomechanical properties of vital spines, these results have to be interpreted with caution.^4^, ^43^, ^44^

Although freezing tissue samples between extraction and testing is common,^45^, ^46^ and freezing spinal segments at −18°C seems not to influence the elastic properties of intervertebral discs during extension, flexion, and lateral bending,^47^ tissue damage while freezing or thawing could not be ruled out completely. Another limitation is the small sample size, which may have created a Type II statistical error and may have produced false negative differences in passive spinal motion during rotation, preventing detection of differences among the three test series.

Regarding the failure tests, statistical analysis is not possible because only one native spinal segment and one segment with two PLCs were tested. Examination of a segment with a single PLC could have provided additional important information on spinal stability following PLC implementation. Further studies are therefore warranted to better determine the failure load of canine lumbar spinal segments after PLCs. The effects on canine lumbar spine biomechanics of performing more than two consecutive PLCs or alternating the side of the PLC should be investigated.

To summarize this study's results, the application of two consecutive right‐sided PLCs to a lumbar spinal segment encompassing two adjacent intervertebral spaces increased passive spinal ROM in the sagittal and dorsal planes. This increase reflected the destabilizing effect of a PLC. However, the biomechanical effect of the second PLC was not substantially greater than that of the first. Given this finding, and considering that positive clinical outcomes have been reported after performing multiple PLCs,^2^, ^8^, ^13^, ^48^ performing two PLCs remains justifiable and potentially beneficial in cases of neurological deficits attributable to protrusions of two consecutive intervertebral discs. Nevertheless, surgeons should exercise caution when performing multiple PLCs and limit bone removal to the minimal effective amount to avoid adverse consequences, including complete spinal failure, luxation, or minor injuries such as repetitive concussive damage to the spinal cord under physiological load.

## AUTHOR CONTRIBUTIONS

Lisa F Becker: Collecting, preparing and embedding the lumbar spinal segments; data acquisition; data analysis and data interpretation; drafting and revising the manuscript. Robin Heilmann, Dipl. Ing.: Adapting the spine testing bench to the needs of the study; performing the biomechanical testing; data acquisition; data analysis and data interpretation. Stefan Schleifenbaum, MSc: Adapting the spine testing bench to the needs of the study; performing the biomechanical testing; data acquisition; data analysis and data interpretation. Stefan Kohl: Implementation of computed tomography. Thomas Flegel, DECVN, DACVIM (Neurology): Guidance in elaborating the study design; guidance in study execution and supervision; revision of the manuscript.

The authors would like to thank Elena Riemer, Dr. Dirk Hasenclever, Lynnda Curry, Monica Solem, Ingmar Kiefer and Susanne Ludewig for technical assistance.

## FUNDING INFORMATION

The study was fully financed by Gesellschaft zur Förderung Kynologischer Forschung e.V.

The results of this study were presented at the 34th Symposium of the European Society of Veterinary Neurology–European College of Veterinary Neurology (ESVN–ECVN) in Palma de Mallorca, Spain, on September 23–24, 2022; at the 10th Leipzig Doctoral Forum (Leipziger Doktorandenforum) in Leipzig, Germany, on July 13, 2023; and at the 2023 German Veterinary Association (DVG) Veterinary Congress in Berlin, Germany, on November 22–25, 2023.

## CONFLICT OF INTEREST

The authors declare no conflict of interest related to this study.
